# Biomarkers for Bladder Cancer Diagnosis and Surveillance: A Comprehensive Review

**DOI:** 10.3390/diagnostics10010039

**Published:** 2020-01-13

**Authors:** Rui Batista, Nuno Vinagre, Sara Meireles, João Vinagre, Hugo Prazeres, Ricardo Leão, Valdemar Máximo, Paula Soares

**Affiliations:** 1 i3S–Instituto de Investigação e Inovação em Saúde, 4200-135 Porto, Portugal; rbatista@ipatimup.pt (R.B.); nunomrvinagre@gmail.com (N.V.); sarameireles@hotmail.com (S.M.); jvinagre@ipatimup.pt (J.V.); hprazeres@i3s.up.pt (H.P.); vmaximo@ipatimup.pt (V.M.); 2 Institute of Molecular Pathology and Immunology of the University of Porto-IPATIMUP, 4200-135 Porto, Portugal; 3 U-Monitor Lda, 4200-135 Porto, Portugal; 4 Faculty of Medicine, University of Porto, 4200-319 Porto, Portugal; 5 Department of Pathology, Faculty of Medicine, University of Porto, 4200-319 Porto, Portugal; 6 Department of Oncology, Hospital Centre of S. João, 4200-319 Porto, Portugal; 7 Pathology Service, Portuguese Institute of Oncology Francisco Gentil (IPO-Coimbra), 3000-075 Coimbra, Portugal; 8 Urology department, Hospital de Braga, 4710-243 Braga, Portugal; romaoleao@gmail.com; 9 Urology department, Hospital CUF Coimbra, 3000-600 Coimbra, Portugal; 10 Faculty of Medicine, University of Coimbra, 3000-370 Coimbra, Portugal

**Keywords:** bladder cancer, biomarker, *TERT* promoter mutation, urinary test, blood test, non-invasive test

## Abstract

Bladder cancer (BC) ranks as the sixth most prevalent cancer in the world, with a steady rise in its incidence and prevalence, and is accompanied by a high morbidity and mortality. BC is a complex disease with several molecular and pathological pathways, thus reflecting different behaviors depending on the clinical staging of the tumor and molecular type. Diagnosis and monitoring of BC is mainly performed by invasive tests, namely periodic cystoscopies; this procedure, although a reliable method, is highly uncomfortable for the patient and it is not exempt of comorbidities. Currently, there is no formal indication for the use of molecular biomarkers in clinical practice, even though there are several tests available. There is an imperative need for a clinical non-invasive testing for early detection, disease monitoring, and treatment response in BC. In this review, we aim to assess and compare different tests based on molecular biomarkers and evaluate their potential role as new molecules for bladder cancer diagnosis, follow-up, and treatment response monitoring.

## 1. Introduction

Bladder cancer (BC) is the sixth most prevalent cancer in both genders and the fourth in males worldwide (incidence of 9.6 and 2.4 per 100,000 in men and women, respectively; age-standardized rates). In 2018, more than half a million people were diagnosed with BC and 200,000 died from the disease. The region with the highest incidence of this cancer was Southern Europe with 15.2 per 100,000 and North Africa with the highest mortality rate of 4.4 per 100,000. Overall, the mortality rate of BC in 2018 was 1.9 in 100,000 [1,2].

The majority of BC arises from epithelial cells and approximately 90% are urothelial tumors, with squamous and glandular-type tumors as less frequent histologic subtypes; more rarely, bladder tumors arise from mesenchymal cells [3]. The most well-established risk factor for BC development is tobacco smoking and it is considered its leading cause. Christensen and collaborators [4] described that cigarette, cigar and pipe smokers had an elevated risk (Hazard ratio (HR): 4.06, 95% confidence interval (CI), 3.84–4.2; HR: 1.61, 95% CI, 1.11–2.32; HR: 1.58, 95% CI, 1.05–2.38, respectively) of dying from a tobacco-related cancer, including bladder cancer. Moreover, cigarette smoking correlates with increased metastasis frequency in pancreatic, breast, and bladder cancer. Several studies have shown that tobacco chemicals can modulate and modify the cell cycle, inducing uncontrolled cell proliferation, through activation of genetic and epigenetic pathways and increasing the expression of proteins involved in inflammation. These altered pathways can be opportunities for the development of new biomarkers and targeted therapies toward the specific molecules involved [5]. High levels of Hypoxia-inducible factor 1 alpha (HIF-1α) expression, caused by chronic hypoxia in chronic obstructive pulmonary disease (COPD), were associated with a higher clinicopathological stage and histological grade in BC and described as independent prognostic variables for overall survival, disease-specific survival, and progression-free survival. The level of HIF-1α expression was an independent prognostic variable for progression-free survival. COPD was referred to as an independent prognostic variable for BC, contributing to poor prognosis [6]. Pezzuto and collaborators confirmed that smoking cessation is a crucial therapeutic option in mild COPD, improving lung function and respiratory symptoms and therefore improving quality of life and minimizing BC risk for these patients [7].

Industrial exposure to aromatic amines, polycyclic aromatic and chlorinated hydrocarbons, long-term use of analgesics, heavy long-term exposure to cyclophosphamide, infection with *Schistosoma haematobium* (an important risk-factor in endemic areas, namely in North Africa), and radiation of the pelvis are also risk factors for chronic inflammation and BC incidence [8].

### 1.1. Classification, Staging, and Grading

In 1973, the first classification of urothelial tumors divided these tumors into three grades: G1 as a low-grade tumor, G3 as a high-grade tumor, and G2 as an intermediate grade tumor between G1 and G3 [9]. This classification was updated in 2004 and later in 2016 with the reclassification of tumors directly into a clearer grading system characterized by low-grade lesions, composed by G1 and part of the lesions previously characterized as G2; and high-grade lesions, encompassing “more aggressive” G2 and previously classified G3 lesions [9]. Also, a new concept was introduced, the papillary urothelial neoplasm of low malignant potential (PUNMLP), characterizing abnormal growth lesions that did not form a tumor, with low malignant potential, that was categorized in the previous grading system as G1.

**Figure 1:**
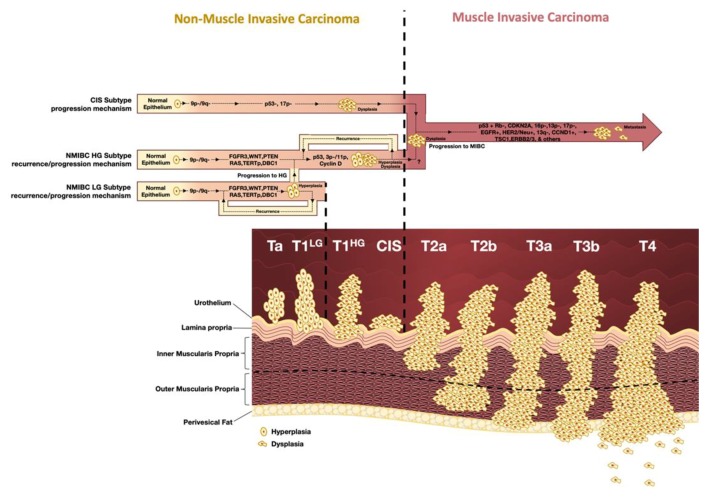
Molecular and histological progression of bladder cancer (BC). Bladder cancer is pathologically characterized by two distinct subtypes—non-muscle invasive carcinoma (NMIBC) and muscle invasive carcinoma (MIBC)—depending on whether it does not or does invade the bladder muscle layer, respectively.

The World Health Organization (WHO) grading system of 2016 stratified non-invasive urothelial tumors as pTa and pT1 in accordance with the invasion of the lamina propria (Figure 1). They are referred to as low-grade (LG) tumors or high-grade (HG) tumors according to cellular features. Carcinoma in situ (CIS) is a non-muscle invasive (NMI) high-grade tumor that is present frequently as a focal or multifocal flat lesion. An interesting and controversial fact is that, although this subclass of tumors is classified as high-grade non-muscle invasive carcinoma (NMIBC), its associated risk to the patient and its molecular features are similar to muscle invasive carcinoma (MIBC) [9,10]. Muscle-invasive cancers (MIBC) are classified as HG tumors and are defined by extension through the bladder muscle layer. The depth of invasion will determine the prognosis and management of the disease and they are classified from T2 to T4, depending on the degree of invasion of the muscular layer, invasion of perivesical tissue or invasion of adjacent tissues/organs [9].

Around 75% to 85% of patients with BC present NMIBC and most of them are stage pTa (70%), followed by pT1 (20%) and CIS (10%). Up to 80% of patients with NMIBC will have at least one recurrence, while around 30% will have disease progression into MIBC (Figure 1) [11].

### 1.2. Clinical Presentation and Management

The majority of BC patients present at diagnosis a painless macroscopic hematuria. Storage symptoms of the low urinary tract, such as dysuria, frequency or urgency in urination can also be detected, mainly in CIS [10,12,13]. More advanced tumors may lead to upper tract obstruction and pain. A complete physical examination, transabdominal ultrasound, and cystoscopy should be performed in patients presenting these symptoms. Urinary cytology may also be considered at the time of cystoscopy.

In case of a suspected lesion in cystoscopy, a transurethral resection of the bladder tumor should be scheduled to confirm the diagnosis and evaluate the extension of the disease. At the moment, the use of urine-based molecular tests as a diagnostic or follow-up tool it is not yet recommended, although such an option is considered promising in the current NMIBC Guidelines [10].

The treatment options available for BC depend on risk stratification and the degree of muscle invasion. The transurethral resection of the bladder (TURB) is the usual management strategy for patients’ diagnosis and treatment of low-grade NMIBC [14]. Immediate intravesical chemotherapy (usually mitomycin C) within 24 h of TURB was shown in some phase III clinical trials to decrease recurrence in selected patients [15,16,17]. Further treatment with intravesical induction (adjuvant) is advised for tumors with an intermediate or high risk of progression. Bacillus Calmette–Guérin (BCG) therapy following TURB shows superiority over TURB alone or TURB and chemotherapy in preventing recurrences of high-grade Ta and T1 tumors [18,19]. Intravesical BCG protocol is instilled intravesically in an induction course (two and four weeks after TURB) composed of six weekly instillations followed by a maintenance course (three weekly instillations at 3, 6, 12, 18, 24, 30, and 36 months) [20].

There is controversy surrounding maintenance of intravesical therapy; however, evidence supports a reduction of disease recurrence and delay of progression with BCG maintenance compared to induction treatment alone [21,22,23,24]. Unfortunately, a significant number of patients are BCG unresponsive and significant ongoing clinical trials reported promising results with cytotoxic, target therapies and immunotherapeutic agents for this important population of patients.

The follow-up for patients with NMIBC (pTa and pT1 high-grade, and CIS) includes urinary cytology and cystoscopy at three to six-month intervals for the first two years, and at six months thereafter until five years, and then yearly [20,25]. Imaging of the upper tract should be considered every one to two years for high-grade tumors [25].

MIBC tumors are either treated with radical cystectomy, associated or not with systemic chemotherapy with platin-based combination regimens, or radiotherapy in selected cases [12,13]. Around 50% of MIBC cases may present micrometastasis at the time of surgery [26,27] and in this setting a cisplatin-based neoadjuvant chemotherapy is the standard of care, presenting an improvement in recurrence-free survival (RFS) and overall survival (OS) in two large randomized clinical trials and in one meta-analysis [28,29]. In a metastatic setting, immunotherapy with checkpoints inhibitors (ICIs) directed to programmed cell-death protein 1 (PD-1) or its ligand (PD-L1) were approved by the US Food and Drug Administration in 2016 for use on patients who are nonresponsive to platinum therapy [30]. Currently, the ICIs in first-line are restricted to selected patients with PD-L1 positivity, advanced BC, and are platinum ineligible [31].

### 1.3. Molecular Subtypes of Bladder Cancer

At the cytogenetic level, NMIBC is genomic stable, usually with a diploid karyotype with few structural genomic rearrangements. It is thought that the loss of chromosome 9 is an early event in the pathogenesis of BC transversal to both NMIBC and MIBC subtypes [32]. MIBCs are frequently aneuploid with several chromosomic rearrangements, rendering them genetically unstable [32]. Molecular fingerprint of BC presents alterations in genes from several pathways, mainly mutations in genes of the cell cycle, chromatin regulation, and tyrosine-kinase signaling [32,33].

In NMIBC, loss of 9p/9q chromosomal arms, *FGFR3* mutations, *TERTp* mutations, and alterations in *WNT*, *PTEN* and *DBC1* is frequent. *RAS* gene mutations are often mutually and exclusively to *FGFR3* mutations. This is rather expected since rat sarcoma virus human homolog (*RAS*) is a downstream effector of Fibroblast growth factor receptor 3 (*FGFR3*) being redundant in the activation of both genes in the signaling cascade. The genetic onset of this subgroup is often identified as “*FGFR3/RAS* type” onset [34,35]. The CIS subgroup, with a more aggressive histologic pattern, is characterized by the early presence of *TP53* alterations [36]. This causes cell dysplasia and when paired with the loss of 17p arm, promotes a quick progression to a full MIBC phenotype. This tumorigenic mechanism is often referred as a “*p53* type” pathway [37]. *FGFR3* alterations are rare in CIS and MIBC, with the apparent exception of the designated luminal MIBC that appears to be enriched with *FGFR3* activating mutations [38].

Thus, either the initial tumor begins as a “*FGFR3/RAS*” or “p53 type” tumor. It can recur as a NMIBC retaining the same alterations, stage, and grade, or progress into MIBC through the acquisition of multiple additional alterations such as the loss of *Rb1* gene, acquisition of *CDKN2A* mutations, loss of 16p, 13q/p, or 17p, and gain of Epidermal growth factor receptor (EGFR) function, among others. These alterations are associated with a worse prognosis [32].

Amplifications of cell cycle genes are also present, particularly Cyclin D1 (*CCND1)*, which is the most common amplified gene in BC, and encodes cyclin D1 [33].

Genetics of BC have been thoroughly characterized in the last 10 years, but the information has not yet been translated into clinical practice in a robust way, mainly for NMIBC. Several biomarker-based tests were approved by the U.S. Food and Drug Administration (FDA) to be used in BC diagnosis, but most of them have an unsatisfactory sensitivity and specificity which leads to a limited use in clinical settings (Table 1). In the present study, we aim to critically review available biomarkers in BC, with an emphasis on NMIBC, as well as identify some emerging molecules or non-invasive tests that are interesting for the diagnosis, prognosis, and/or follow-up of patients with BC.

## 2. Method of Search

A systematic literature search was performed using PubMed, Science Direct, Scopus, Scholar Google, and Russian Google databases to identify studies and reports on bladder cancer established biomarkers and potential biomarkers using keywords such as “bladder cancer”, “biomarker”, “non-muscle invasive bladder cancer”, and “non-invasive”. Only literature discussing human studies was considered.

## 3. Biomarkers for Bladder Cancer Diagnosis and Follow-up (Currently Available FDA-Approved Tests and Kits)

Bladder cancer follow-up presents several caveats. More than 2/3rd of NMIBC patients recur after primary tumor removal. The long-life recurrence follow-up program in these patients through an extremely invasive procedure, cystoscopy, emphasizes this problem.

The intensive follow-up program, through cystoscopy and urinary cytology, makes bladder cancer the most expensive follow-up, in comparison to other cancers [39,40]. Urine cytology is the classical non-invasive test in the diagnosis and follow-up of BC patients, used as a gold standard together with cystoscopy. This test is non-invasive, easy to perform, non-expensive, and can achieve a specificity up to 98% in high-grade tumor detection. However, urinary cytology has an overall low sensitivity (less than 40%), discouraging the persistent use in primary evaluation of bladder cancer suspicion as a diagnostic tool [41]. Regardless of the limitations, voided urine cytology is still included as the standard of care as an adjunct to cystoscopy to detect high-grade tumors. However, paradigm is changing towards the use of new methodologies in the follow-up of patients through cost-effective, non-invasive procedures that will help surpass the current limitations of BC follow-up. The follow-up of NMIBC patients can greatly benefit from non-invasive tests that not only allow screening of recurrence, but also predict response to therapy.

**Figure 2:**
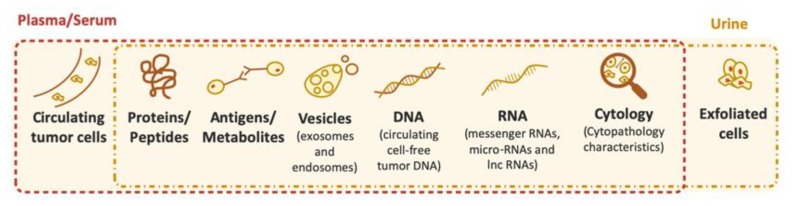
Non-invasive targets and starting material.

Numerous studies are being develop in order to enlarge the diagnostic accuracy of urinary tests and the creation of alternatives to cytology and/or cystoscopy. A vast quantity of potential biomarkers are described in the literature aiming to detect genomic, transcriptomic, epigenetic or protein changes in serum or in urine samples (Figure 2) [42]. Some of these tests underwent clinical validation and obtained FDA approval. From the FDA-approved tests, urinary cytology, nuclear matrix protein 22 (NMP22) kit, NMP22 *BladderChek* Test, *BTA-TRAK* and *BTA stat* kits, *Cell Search*, and *UroVysion* are approved for initial detection and surveillance of BC whereas some tests, such as *uCyt +*, are only approved for the follow-up of BC [43]. Despite not having FDA approval, other tests exist, and some with European Conformity Approval (CE) marking such as *Epicheck* and *Uromonitor*, present promising sensitivity and specificity values. All these tests contribute to a diverse choice and demonstrate the efforts to generate a test that will improve patients’ life quality. A brief description of the FDA- and CE-approved tests and values for the sensitivity and specificity presented by the manufactures of each kit are available in Table 1.

### 3.1. NMP-22 Protein Test

Nuclear matrix protein 22 (NMP22) is a nuclear matrix protein involved in the distribution of chromatin during mitosis [44]. It is generally elevated in BC but can be present in normal urothelial cells. NMP22 detection tests are either based on a quantitative ELISA or a quantitative point-of-care test (*BladderChek*). In patients presenting microscopic hematuria (three or more erythrocytes per field) the use of the NMP22 test was more sensitive for BC detection (70% of sensitivity) in comparison with cytology alone (27% of sensitivity). Its performance in diagnosis varies with tumor grade, with a lower sensitivity in the detection of low-grade lesions [44,45]. Due to the presence of NMP22 in normal urothelial cells, false-positive results are expected and impair diagnostic specificity; the NMP22 test has a lower specificity in comparison with cytology, 80% and 96%, respectively [45]. In follow-up of BC patients, NMP22 has a low sensitivity (33%), nonetheless higher than cytology (25%), and NMP22 specificity was also lower than cytology (76% and 97%, respectively) in that setting [45].

### 3.2. BTA Stat and BTA-TRAK

There are two available tests that target human complement factor-H related protein (hCFHrp), *BTA stat* and *BTA-TRAK*. *BTA stat* (Polymedco Inc., Cortlandt Manor, NY, USA) is a point-of-care test based on an immunochromatographic assay for the detection of hCFHrp in urine from patients previously diagnosed with BC. Bladder Tumor Antigen (BTA) is produced by bladder tumor cells and not by any other epithelial cell lines [46]; it is released into the urine of patients with bladder cancer as the tumor invades the stroma [47]. The test can be performed in a few minutes, without any pre-treatment of the urine sample, thus rendering it as very practical [48]. It is approved for follow-up of NMIBC patients. A study by Raitanen et al. evaluated the potential use of *BTA stat* for follow-up and verified an overall sensitivity superior to cytology (56.0% vs. 19.2%) but with a lower specificity (85.7% vs.98.3%). On a series of 194 patients with BC and 185 controls, *BTA stat* was more sensitive than cytology with an increased sensitivity with higher tumor stage. The values of sensitivity (73.6%) and specificity (83.3%) in this diagnostic cohort were similar to the Raitanen et al. study [49]. It is important to point out that both studies had false-positive results associated with inflammation, cystitis, tuberculosis, and past BCG instillations, that decreased the specificity of this test [48,49].

*BTA-TRAK* is a urine ELISA that also detects hCFHrp. In a multicenter study, *BTA-TRAK* showed superior sensitivity in comparison with cytology (66% vs. 33%) but lower sensitivity than *BTA stat*, and a higher sensitivity for high-grade tumors (74%) than low-grade tumors (25%). *BTA-TRAK* was more sensitive than cytol ogy in the detection of low-grade tumors, such as Ta and T1 tumors. As expected, cytology had a superior specificity than *BTA-TRAK* (99% vs. 69%) [50].

### 3.3. UroVysion

*UroVysion* (Abbott Molecular, Chicago, IL, USA) is a urine test based on fluorescent in situ hybridization (FISH), identifying common chromosomal abnormalities present in BC, including alterations in chromosomes 3, 7, and 17, and the most common alteration, deletion of 9p21 [51]. It requires the use of FISH probes and visualization of the results under a fluorescent microscope rendering the test particularly expensive to perform and requiring highly specialized equipment and personal. Other primary tumors, such as of the renal pelvic, ureteral transitional cell carcinoma, prostatic carcinoma, and others, can present chromosomal aberrations targeted by *UroVysion* and may result in a positive urine *Urovysion* result rendering it not specific in the detection of BC. A study comparing *UroVysion* with cytology showed that *UroVysion* had higher sensitivity (62% vs. 29%) but lower specificity (89% vs. 97%). The authors recommended that *UroVysion* should be used only in high-risk patients, particularly in those patients who have an equivocal urine cytology [52]. Another study with 129 urine specimens comparing *UroVysion* and cytology showed a sensitivity of 67% and 69%, and a specificity of 72% and 76%, respectively. Importantly, tumors that were missed by cytology were also not detected by *UroVysion* [53].

### 3.4. ImmunoCyt/uCyt+

*ImmunoCyt/uCyt+* is the only commercially available kit used for BC follow-up. This test uses fluorescent-labeled antibodies to three proteins (carcinoembryonic antigen (CEA); two mucins, LDQ10 and M344) detected on malignant exfoliated urothelial cells. A longitudinal study evaluated *uCyt+* and cytology performance for low-grade tumor (pTa G1-2) follow-up. As other tests, *uCyt+* specificity was affected by the presence of hematuria, decreasing values from 82% to 67%. According to the authors, if *uCyt+* was used as a follow-up tool, 75% of cystoscopies would be avoided [54].

Overall, *BTA stat, ImmunoCyt,* and *Urovision* present higher sensitivity when used as diagnosis and follow-up tools in comparison with urinary cytology but present a lower specificity. Due to their low sensitivity in the detection of low-grade tumors, it is suggested that these biomarkers might be more useful in the follow-up of high-risk tumors [55].

### 3.5. Uromonitor and Uromonitor-V2

The *Uromonitor* test kit (U-Monitor, Porto, Portugal) is a procedure developed and optimized for the detection of hotspot mutations in *TERTp* and *FGFR3* genes in DNA from tumor cells exfoliated to urine in a real-time PCR platform. This test relies on a novel sample processing and conservation system, through a urine filtering system allowing sample pre-processing and conservation until analysis. Screening of targeted alterations is based on a highly sensitive real-time allelic discrimination assay using lock nucleic acid (LNA) competitive probes for *TERTp* alterations and a modified competitive allele-specific real-time detection PCR for *FGFR3* alterations. The use of the custom method improved the current detection limit for these alterations when compared to Sanger sequencing, enhancing the ability to detect a minimal quantity of altered cells in a large pool of cells without alterations, and improved the cost and time response compared to next generation sequencing (NGS)-based methods.

In a multicentric validation study, *Uromonitor* presented a sensitivity of 73.5% in the detection of TURB confirmed recurrence, with a specificity of 73.2% and overall good performance across stage and grade [56]. The values were comparable and similar to gold standard cystoscopy performance that presented values of 79.4% and 73.2% for sensitivity and specificity, respectively. Compared with cytology, *Uromonitor* presented higher sensitivity than cytology (42.9% cytology sensitivity vs. 73.5% *Uromonitor*), and when combined with cystoscopy achieved 100% sensitivity and 88.6% specificity, an important upgrade in sensitivity and specificity compared with cystoscopy + cytology combination (sensitivity of 86.7%). [56]. *Uromonitor* used with cystoscopy at a routine level would lead to a cost-effectiveness increment and might be used in follow-up with better performance relative to the current cytology, when cystoscopy cannot be performed or is not routinely available. In low-grade recurrence-positive patients, *Uromonitor per se* achieved a 62.5% detection rate, while in patients that recurred with a high-grade tumor, *Uromonitor* tumor detection rate was 75%. The presence of inflammation or other benign lesions in the urinary tract did not seems to affect the sensitivity and specificity of *Uromonitor test*.

*Uromonitor-V2* added Kirsten rat sarcoma viral oncogene homolog (KRAS) hotspot detection to the *Uromonitor* kit. The *Uromonitor-V2* test presented in a multicenter study achieved 100% sensitivity, 83.3% specificity, a Positive Predictive Value (PPV) of 66.7% and a Negative Predictive Value (NPV) of 100% among 122 NMIBC patients undergoing “conventional” surveillance for NMIBC [56].

### 3.6. UroSEEK

*UroSEEK* is a non-invasive molecular-based test, aimed at detecting BC through the analysis of tumor DNA present in the urine, by NGS, and targeting ten typical mutations of BC, namely *FGFR3*, *TP53*, *ERBB2*, *CDKN2A*, *KRAS*, *HRAS*, *MET*, *PIK3CA*, *MLL,* and *VHL* and Sanger sequencing of *TERTp* alterations [65,66]. The main focus of this test is not to substitute cytology but rather be complementary with it. *UroSEEK* was more sensitive than cytology in the surveillance cohort (71% vs. 25%) and in the primary detection cohort (95% vs. 43%). In line with other tests, the specificity of cytology was superior to this technic on the detection cohort (100% vs. 93%). *UroSEEK* was also effective in detection of cases where the cytology was atypical, predicting the progression of 95% of patients with atypical cytology that developed BC [65].

In another study, *UroSEEK* presented concordant results, reaching in the early-detection cohort a sensitivity of 96% and a specificity of 88% with an NPV of 99%. In a disease surveillance context, *UroSEEK* presented a poorer performance. *UroSEEK’s* sensitivity of 74%, specificity of 72%, and NPV of 53%, seemed to be in accordance with the prior described study [66]. From the aforementioned studies, *UroSEEK* could potentially be used in the diagnosis or first approach of patients who have suspicion of having BC or with an atypical urinary cytology. As a drawback, this test does not present an excellent performance in the follow-up of patients with prior diagnosis of BC. Although it is an NGS-based technique, the screening of *TERTp* mutations, the most frequent alterations in NMIBC, is based on Sanger sequencing, a technique known to present low sensitivity in the detection of trace amounts of mutation, hence impairing the sensitivity. Like other kits, *UroSEEK* does not distinguish the upper tract urothelial carcinomas.

### 3.7. EpiCheck

Methylation and other epigenetic changes are important in the diagnosis and follow-up of BC patients. *EpiCheck* is currently the only developed product to take advantage of these types of biomarkers.

*EpiCheck* is a urine-based assay that analyses 15 DNA methylation markers commonly altered in BC aiming to detect the presence of recurrence. The analysis is performed using a probability algorithm developed by EpiScore, providing a probability (range 0 to 100) that the test is detecting BC. On a multicenter and prospective study of 353 patients with NMIBC on follow-up, *EpiCheck* presented a sensitivity of 68.2% and a specificity of 88.0%, with an NPV of 95.1%. *EpiCheck* was more effective when analysis excluded low-grade recurrences, achieving an area under the curve (AUC) of 0.94. This data supports the claim that this assay could be used on the follow-up of patients, since the likelihood of detecting a high-grade tumor would be substantially higher [64]. *EpiCheck* presented higher sensitivity with a lower specificity when compared with cytology (62.3% vs. 33.3% and 86.3% vs. 98.6%, respectively). Similarly, *EpiCheck* was more sensitive in high-grade tumors, where the obtained NPV in this study was 82.9% [67]. As a pitfall, *EpiCheck* is costly and technically challenging in execution, but unlike other tests, the presence of inflammation in the urinary tract did not affect the sensitivity and specificity of *EpiCheck* [64,67].

## 4. Other Biomarkers for Bladder Cancer Diagnosis and Follow-up

Although a reasonable number of non-invasive options are available in the market for the diagnosis and follow-up of BC, other potential biomarkers are emerging to be applied in the future, in the non-invasive detection of BC.

### 4.1. TERTp Mutations and Hypermethylation

In recent years, telomerase reverse transcriptase (TERT) genetic events gained relevance in BC due to their potential to be used as excellent biomarkers in both follow-up and new diagnosis.

*TERTp* mutations located at -124 and -146 base pairs (bps) upstream of the transcription of *TERT* gene coding region, are the most common mutations in BC and apparently an early event on oncogenesis [68,69]. *TERTp* mutations emerged as a novel biomarker detected in up to 80% of BC, independently of stage or grade [70,71,72,73] and surpassed the frequency of *FGFR3* mutations in NMIBC [74,75]. The transversal genetic profile strongly suggests its participation in the two major genetic pathways of urothelial tumorigenesis (NMIBC and MIBC) [71,72]. *TERTp* mutations were a game changer in BC and had the potential to be considered a useful urinary biomarker for disease monitoring and early detection of recurrence, being currently included in tests *Uromonitor, Uromonitor-V2,* and *UroSEEK*. *TERTp* mutations are not present in inflammatory or urinary infections, which is a current pitfall in some of the non-invasive assays [71,72,76,77].

Also targeting the *TERT* gene promoter, aberrant *TERT* promoter methylation is detected in a large number of cancers, in a region described as *TERT* hypermethylated oncological region (THOR). THOR is an alternative telomerase-activating mechanism in cancer and can be a prognostic biomarker [78]. Directly related to BC, THOR hypermethylation and *TERTp* mutations are common and coexist in BC; THOR hypermethylation associates with disease progression, with the combined genetic and epigenetic alterations of *TERT,* bringing additional prognostic value in NMIBC [79].

### 4.2. miRNA Biomarkers

Multiple evidences demonstrated that microRNAs (miRNAs) can act as tumor suppressors or oncogenes in BC. They have an important role in oncogenesis and their use as potential biomarkers should not be disregarded, in particular because miRNAs are also more resistant to degradation by nucleases [80]. Hanke et al. described that BC patients presented higher expression of miR-126, miR-182, and miR-199a in urine. Results showed that miR-126 was the most sensitive and specific for detecting BC, with values of 72% and 82%, respectively [80]. In another study, a panel of two upregulated (miR-152 and miR-148b-3p) and four downregulated (miR-3187-3p, miR-15b-5b, miR-27a-3p, and miR-30a-5p) showed an AUC of 0.956 with a sensitivity of 90% and specificity of 90% for BC detection. miR-152 high levels and miR-3187-3p low levels were statistically associated with advanced tumor stage [81]. miR-101 was described as decreased in the serum of BC patients when compared with healthy controls, presenting a performance with an AUC of 0.884, a sensitivity of 82.0%, and a specificity of 80.9%. This miRNA has also been significantly associated with Tumor/Node/Metastasis (TNM) stage, pathological grade, and lymph node metastasis [82]. miR-10 and miR-138 were evaluated as prognostic biomarkers in a study including BC tissue and its paired adjacent tissue from a cohort of patients with NMIBC at stages Ta/T1. miR-138 presented a significant higher-expression in low-grade and Ta stage tumors and a low expression in tumors from patients that recurred. Survival analyses showed that higher miR-138 expression levels were associated with increased recurrence-free survival whereas patients harboring tumors with low expression of miR-100 presented high progression-free survival (PFS) and improved cancer-specific overall survival (OS) [83]. Another study evaluated the expression levels of miR-205 on plasma of NMIBC patients, MIBC patients, and healthy controls [84]. miR-205 presented an AUC for detection of BC of 0.950, with a sensitivity of 76.4% and a specificity of 96.4%. High expression of miR-205 was associated with a shorter disease-free and disease specific survival in univariate but not multivariate analysis, hence impairing its usefulness as a prognosis biomarker yet retaining its interest as a diagnosis biomarker [84]. Xan et al. described that high levels of miR-155 were associated with lower recurrence-free and PFS. miR-155 expression also distinguished the cluster of patients with BC from the cluster without BC with a sensitivity of 80.2% and a specificity of 84.6% [85]. Evaluation of specific methylation pattern in miR-137, miR-124-2, miR-124-3, and miR-9- in voided urine samples of BC patients with NMIBC presented, for recurrence detection, a 61.5% sensitivity and 74.0% specificity. The highest levels of methylation were associated with recurrence and with a greater likelihood of receiving radical cystectomy [86], further enhancing the usefulness of this type of biomarker in disease follow-up and diagnosis.

### 4.3. lncRNAs Biomarkers

Long-noncoding RNAs (lncRNAs) are important regulators of genetic and epigenetic expression and can interact with miRNAs promoting or repressing its activity [87]. Inevitably, such close interactions with miRNAs enhance the potential of lncRNAs to be used as biomarkers in the same context.

A study evaluating the plasma levels of the lncRNA TUC338 in patients with BC when compared with healthy controls, observed that lncRNA TUC338 was significantly upregulated in early-stages BC patients, with an AUC of 0.9239, when considering an initial diagnosis purpose. This study also disclosed that lncRNA TUC338 acts as an enhancer of miR-10b, that was also elevated in BC patients and its high expression related with a poorer survival, thus reflecting the influence of lncRNAs on the oncogenic pathway of BC. lncRNA TUC338 levels decreased one month after treatment when compared with pre-treatment levels, thus reflecting not only the potential of lncRNAs as a diagnosis biomarker but also as a treatment responsiveness biomarker [88].

Eissa et al. [89] evaluated the expression levels of several miRNAs, lncRNAs, and gene expression in the plasma and voided urine of patients with BC, patients with benign urological conditions, such as benign prostatic hyperplasia or bilharzial cystitis, and healthy controls. BC patients presented higher expression levels of miR-324-5p, miR-4738-3p, and FBJ murine osteosarcoma viral oncogene homolog B (FOSB) mRNA, and lower expression levels of lncRNA miR-497-HG and Regulator of Calcineurin 1 (RCAN1) mRNA on voided urine. When directly compared with cytology that presented an accuracy of 69.9%, the potential biomarker with lowest accuracy was lncRNA miR-497-HG with 78.3% and the highest, with 99.3% accuracy, were FOSB mRNA and RCAN1 mRNA, with a specificity of 98.9%, exhibited more sensitivity than cytology [89].

### 4.4. Extracellular Vesicles

Extracellular vesicles (EVs) are a new attractive biologic sample to BC monitoring since these structures can contain several cellular constituents, such as proteins, DNA or mRNA. Extracellular vesicles present unique features such a low rate of degradation, due to their membrane similarity with the cell of origin, allowing them to overcome detection from the immune system [90]. Liang et al. developed an innovative device for microfluidic filtration aimed at the isolation and enrichment of EVs from urine, within a size range of 30–200 nm, that were subsequently quantified via a microchip Enzyme-Linked Immunosorbent Assay (ELISA). With this device, the authors showed that BC patients had higher concentration of extracellular vesicles in the urine when compared with healthy controls, and detection with this device allowed a sensitivity of 81.3% and a specificity of 90.0% in the discrimination of BC patients against healthy controls [91].

## 5. Predictive Biomarkers for Therapy in Bladder Cancer Patients

BC patients have several treatment options available, though some can be aggressive and affect the morbimortality of those patients. The identification of biomarkers-driven patient stratification could help to make decisions about who will benefit more from particular therapies in different treatment settings, such as intravesical therapy, immune checkpoint inhibitors, and molecular-targeted drugs [92]. Around 30% to 50% of patients fail to respond to intravesical BCG therapy in NMIBC and about half of MIBC patients do not respond to neoadjuvant chemotherapy delaying the chance to be cured by radical surgery [93,94].

New advances in modern sequencing technologies increased the understanding of BC tumor biology and the molecular mechanisms of action of therapies in NMIBC and MIBC. Nevertheless, clinically applicable biomarkers to predict response to therapies in both settings are not yet available.

Clinicopathological characteristics including tumor stage, grade, presence of CIS, tumor size, tumor multiplicity, and recurrence are bona-fide features that most strongly correlate with response to intravesical therapy but are not predictive of treatment outcome in NMIBC [95]. Knowing that cytokine expression is related to positive outcomes, Kamat et al. [96] published the nomogram cytokine panel in response to intravesical therapy (CyPRIT) (IL-2, IL-8, IL-6, IL-Ira, IL-18, IL-12 (p70)), Il-12 (p40), tumor necrosis factor-related apoptosis-inducing ligand, and tumor necrosis factor-β) predicting recurrence following BCG induction, with an accuracy of 85.5%, but requiring validation.

Recent findings concerning immunologic features of BC emerged as a potential predictive tool in response to intravesical BCG and even to immunotherapy with checkpoint inhibitors in more advanced disease. Some studies failed to show a correlation between programmed cell-death protein ligand 1 (PDL-1) expression and outcomes of NMIBC after BCG induction, with conflicting results and no conclusive evidence [97]. However, evaluation of BCG-specific T-cell immunity can improve the outcome of BCG immunotherapy [98,99]. The T-cell and MDSC ratio in urine before and after BCG treatment was also related with BCG failure [100].

Some authors have tried to screen for mutations that can be linked with BCG response. One example is AT-rich interactive domain-containing protein 1A (*ARID1A)* mutation that was associated to a higher risk of BCG unresponsiveness [101]. We also observed that specific *TERTp* genotypes can be associated to the response to BCG (unpublished data).

Other authors [102] are trying new approaches in the development of mutation driven cancer biomarkers based on the dynamic process of cancer cells. The authors also describe that mutations, leading to loss-of-heterozygosity can be a consequence of defective DNA damage response mechanisms. These interesting results warrant a possibility for the utilization of this information on tumor identification and possibly improved early diagnosis, staging, and treatment of cancers [102,103].

Perhaps, a combination of tumor characteristics and pre-treatment immune system status could be established as the better way to predict response to BCG therapy. Randomized trials combining therapy with PD-L1 inhibitor and BCG for high-risk NMIBC patients are ongoing and results can be promising on the management of BCG naïve or refractory NMIBC [104,105].

Taking into account the most frequently harbored mutations in BC, further development of molecular biomarkers drug-targetable will hopefully maximize drugs response and improve patient survival.

## 6. Concluding Remarks

Despite high sensitivities and specificities, the now-available non-invasive assays for BC diagnosis and follow-up, still present inconvenient rates of false positive results. False positive rates can arise from several factors, including the presence of benign conditions, as hematuria, cystitis, lithiasis, urinary tract infections, inflammation or even because of repeated instrumentation, such as cystoscopy.

A vast offer is available for the non-invasive detection of BC, with each assay presenting their own advantages and disadvantages. No leading kit is currently being broadly used, regardless, a large part have been on the market for some time. This should not be interpreted as a pitfall of non-invasive methods since most of them present better performance than conventional cytology. These kits are not yet implemented in clinical practice since none of them are fulfilling all the expectations in all points of the detection of BC. Healthcare professionals could take advantage and use each kit according to the specific patient, context, and limitations in the detection of BC. New biomarkers to predict disease recurrence and response to therapy are being developed and could tackle this important biological question that is patient stratifications.

We are on the way towards reliable biomarkers and assays to be used in non-invasive settings, that are needed for earlier diagnosis of BC and early detection of recurrence, particularly in low-grade and low-stage NMIBC. Certainly, in the new era of liquid biopsies and precision oncology, cell-free (cfDNA) analysis in urine and/or serum will potentially overcome these obstacles and successfully validate molecular and immunological biomarkers-driven patient stratification in the clinic.

## Figures and Tables

**Table 1:** Available commercial kits performance and characteristics.

Name (Commercially Available Kits/Procedures)	FDA Approval/CE Mark	Present in EAU Guidelines 2019	Sample	Starting Material	Technology	Type of biomarker assessed	Purpose	Overall Performance	References
**Cytology**	**Yes**	**Yes**	**Urine**	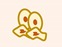 **Exfoliated cells**	**Giemsa an H&E staining**	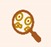 **Cell phenotype**	**Diagnostic & Surveillance**	**Sensitivity = 38%** **Specificity = 98%**	Christopher G.T. Blick, et al. 2011 [41]
**uCyt+**	Yes/NA*^1^	**No**	**Urine**	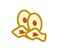 **Exfoliated cells**	**Immunofluorescence**	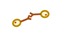 **Antigens/** **Metabolites**	**Surveillance**	**Sensitivity = 73%** **Specificity = 66%**	He, H. et al. 2016 [57]
**NMP22**	**Yes/Yes**	**Yes**	**Urine**	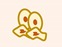 **Exfoliated cells**	**Elisa**	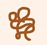 **Peptides**	**Surveillance**	**Sensitivity = 40%** **Specificity = 99%**	Mowatt, G. et al. 2010 [58]
**CellSearch**	**Yes/Yes**	**No**	**Plasma/serum**	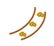 **CTC’s**	**Immunomagnetic enrichment**	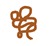 **Proteins**	**Surveillance**	**Sensitivity = 35%** **Specificity = 97%**	Zhang, Z. et al. 2017 [59]
**UroVysion**	**Yes/Yes**	**Yes**	**Urine**	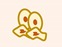 **Exfoliated cells**	**FISH**	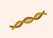 **DNA** **(Aneuploidies)**	**Diagnostic**	**Sensitivity= 72%** **Specificity = 83%**	Hajdinjak, T. et al. 2008 [60]
**BTA stat/** **BTA Track**	Yes/NA*^1^	**No**	**Urine**	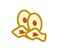 **Exfoliated cells**	**Dipstick immunoassay**	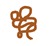 **Proteins**	**Diagnostic & Surveillance**	**Sensitivity = 70%** **Specificity = 75%**	Glas, A.S. et al. 2003 [61]
**CxBladder**	**No/No**	**No**	**Urine**	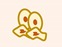 **Exfoliated cells**	**RT-qPCR**	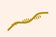 **RNA (messenger RNA)**	**Diagnostic**	**Sensitivity = 82%** **Specificity = 85%**	O’Sullivan, P. et al. 2012 [62]
**Xpert Detection**	**No/Yes**	**No**	**Urine**	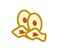 **Exfoliated cells**	**RT-qPCR**	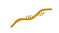 **RNA (messenger RNA)**	**Diagnostic**	**Sensitivity = 76%** **Specificity = 85%**	Valenberg FJP, V. et al. 2017 [63]
**Uromonitor**	**No/Yes**	Yes*^2^	**Urine**	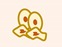 **Exfoliated cells**	**Real-time PCR**	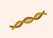 **DNA** **(tumor cell DNA)**	**Surveillance**	**Sensitivity = 74%** **Specificity = 93%**	Batista, R. et al. 2019 [56]
**Epicheck**	**No/Yes**	**No**	**Urine**	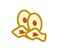 **Exfoliated cells**	**Real-time PCR**	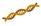 **DNA** **(methylation)**	**Surveillance**	**Sensitivity = 68%** **Specificity = 88%**	Witjes, A. et al. 2018 [64]
**UroSEEK**	**No/No**	**No**	**Urine**	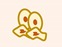 **Exfoliated cells**	**Massively parallel sequencing-based assay**	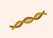 **DNA** **(tumor cell DNA)**	**Diagnostic & Surveillance**	**Sensitivity = 95%** **Specificity = 93%**	Springer, S.U. et al. 2018 [65]

*1 Information not available; *2 EAU guidelines refer the use of TERT/FGFR3 testing that composes Uromonitor test.
